# Prediction of presynaptic and postsynaptic neurotoxins by combining various Chou’s pseudo components

**DOI:** 10.1038/s41598-017-06195-y

**Published:** 2017-07-19

**Authors:** Haiyan Huo, Tao Li, Shiyuan Wang, Yingli Lv, Yongchun Zuo, Lei Yang

**Affiliations:** 1 Department of Environmental Engineering, Hohhot University for Nationalities, Hohhot, 010051 China; 2 College of Life Science, Inner Mongolia Agricultural University, Hohhot, 010018 China; 3 College of Bioinformatics Science and Technology, Harbin Medical University, Harbin, 150081 China; 4 The Key Laboratory of Mammalian Reproductive Biology and Biotechnology of the Ministry of Education, Inner Mongolia University, Hohhot, 010021 China

## Abstract

Presynaptic and postsynaptic neurotoxins are two groups of neurotoxins. Identification of presynaptic and postsynaptic neurotoxins is an important work for numerous newly found toxins. It is both costly and time consuming to determine these two neurotoxins by experimental methods. As a complement, using computational methods for predicting presynaptic and postsynaptic neurotoxins could provide some useful information in a timely manner. In this study, we described four algorithms for predicting presynaptic and postsynaptic neurotoxins from sequence driven features by using Increment of Diversity (ID), Multinomial Naive Bayes Classifier (MNBC), Random Forest (RF), and K-nearest Neighbours Classifier (IBK). Each protein sequence was encoded by pseudo amino acid (PseAA) compositions and three biological motif features, including MEME, Prosite and InterPro motif features. The Maximum Relevance Minimum Redundancy (MRMR) feature selection method was used to rank the PseAA compositions and the 50 top ranked features were selected to improve the prediction accuracy. The PseAA compositions and three kinds of biological motif features were combined and 12 different parameters that defined as P1-P12 were selected as the input parameters of ID, MNBC, RF, and IBK. The prediction results obtained in this study were significantly better than those of previously developed methods.

## Introduction

Neurotoxins can be divided into presynaptic and postsynaptic neurotoxins based on their mechanism of action^1^. Presynaptic neurotoxins are commonly called β-neurotoxins. These neurotoxins act on the plasmatic membranes of nerve endings, promote the generation of interterminal signals, and lead to a massive stimulation of the release of the neuromediator^2–4^. Presynaptic neurotoxins are rich sources of phospholipases^5–9^ and produce neuromuscular blockade by inhibiting the release of acetylcholine from the presynaptic membrane^10^. Postsynaptic neurotoxins are commonly called α-neurotoxins^11–13^, and most of these neurotoxins are from the venoms of snakes of families. Postsynaptic neurotoxins bind specially to the nicotinic acetylcholine receptor resulting in the prevention of nerve transmission, leading to death from asphyxiation^14–17^. Due to postsynaptic neurotoxins have similarity action to the reversible acetylcholine receptor antagonist curare with curare-mimetic toxins, there are often referred to as “curare-mimetic toxins”^5^. These two neurotoxins contribute to the understanding of the molecular steps of neurotransmission, and have potential use in cell biology and neuroscience research as well as therapeutics in some human neurological disorders. For example, presynaptic neurotoxins have been used for the treatment of migraine headache and cerebral palsy^18^. With the numerous of neurotoxin sequences generated in the post-genomic era, it is desired to develop a method for identification of neurotoxins for basic research and drug discovery.

In recent years, many computational algorithms have been developed for analyzing and predicting toxins. Short animal toxin and toxin-like protein sequences can be predicted by the web-based classifier ClanTox^19, 20^. The neurotoxins and bacterial toxins derived from Swiss-Prot were predicted by Feed-forwarded Neural Network (FNN), Partial Recurrent Neural Network (RNN) and Support Vector Machine (SVM)^21–23^. Four kinds of conotoxin superfamilies for 116 conotoxin sequences were predicted by ISort predictor, Least Hamming, Multi-class SVMs, one-versus-rest SVMs^24^, modified Mahalanobis discriminant^25^, and dHKNN^26^. Four conotoxin superfamilies for 261 conotoxin sequences that collected from Swiss-Prot were predicted by SVM^27^. In our previous work, based on the Animal Toxin Database (ATDB)^28, 29^, the presynaptic and postsynaptic neurotoxins were predicted by Increment of Diversity (ID)^30^, and the correlation coefficient (CC) value was 0.7963 when evaluated by the jackknife test.

In this study, four algorithms were proposed for predicting presynaptic and postsynaptic neurotoxins by using Increment of Diversity (ID), Multinomial Naive Bayes Classifier (MNBC), Random Forest (RF), and K-nearest Neighbours Classifier (IBK). Pseudo amino acid (PseAA) compositions, MEME motif features^31^, Prosite motif features^32^ and InterPro motif features^33^ were used to represent the protein sequences. The Maximum Relevance Minimum Redundancy (MRMR)^34, 35^ was used to rank the features for improving the performance of the predictors. When these algorithms were applied to the neurotoxin dataset with 78 presynaptic neurotoxins and 69 postsynaptic neurotoxins, the overall success rates obtained by the jackknife test were significantly higher than those of existing classifier on the same dataset. In addition, as demonstrated by a series of recent publications^36–43^ in compliance with Chou’s 5-step rule^44^, to establish a really useful sequence-based statistical predictor for a biological system, we should follow the following five guidelines: (a) construct or select a valid benchmark dataset to train and test the predictor; (b) formulate the biological sequence samples with an effective mathematical expression that can truly reflect their intrinsic correlation with the target to be predicted; (c) introduce or develop a powerful algorithm (or engine) to operate the prediction; (d) properly perform cross-validation tests to objectively evaluate the anticipated accuracy of the predictor; (e) establish a user-friendly web-server for the predictor that is accessible to the public. Below, we are to describe how to deal with these steps one-by-one.

## Results

### Phylogenetic trees of presynaptic and postsynaptic neurotoxins

**Figure 1:**
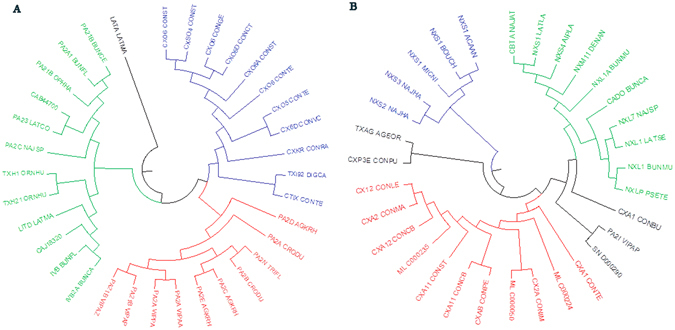
The phylogenetic trees for (**A**) presynaptic neurotoxins and (**B**) postsynaptic neurotoxins.

In this study, the Molecular Evolutionary Genetics Analysis (MEGA) software^45^ was used to provide the phylogenetic trees of presynaptic and postsynaptic neurotoxins, only the neurotoxins that had the signal peptides were uploaded to the MEGA software for generating phylogenetic trees. The phylogenetic trees for presynaptic and postsynaptic neurotoxins were shown in Fig. 1A and B, respectively. These two figures illustrated some useful information about the inferred evolutionary relationships among those two neurotoxins, and the neurotoxins that in the same branch were believed to have a common ancestor. The Fig. 1A and B may also help us to better understand how the presynaptic and postsynaptic neurotoxins diversified over times.

### Analysis of Prosite motif features

In 78 presynaptic neurotoxins, PS00118 was conserved in 29 sequences and PS00119 was conserved in 31 sequences. PS00118 is a pattern of phospholipase A2 histidine active site which is centered on the active site histidine and PS00119 is a pattern of phospholipase A2 aspartic acid active site which is centered on the active site aspartic acid. Both PS00118 and PS00119 contain three cysteines that involved in disulfide bonds. PS60004 belongs to PROSITE documentation PDOC60004 which is a pattern of omega-conotoxin family signature, and appears in 19 presynaptic neurotoxins. Omega conotoxins are calcium channel blockers and the cysteine arrangement [C-C-CC-C-C] is included in PS60004. PS00280, PS01138, PS01186, PS60015, PS60021, PS60022, PS60023 and PS60025 are also observed in presynaptic neurotoxins. PS00272 is a pattern of snake toxin signature and observed in 49 sequences. Snake toxins are a group of short and long neurotoxins, cytotoxins, short toxins and miscellanous venom peptides. Snake toxin signature includes four conserved cysteines and a conserved proline is thought to be important for the maintenance of the tertiary structure. The second cysteine in this pattern is linked to the third cysteine by a disulfide bond. PS60014 is a pattern of alpha conotoxin family signature and appears in 8 postsynaptic neurotoxins. This pattern includes a common part of the cysteine arrangement [CC-C-C], four conserved cysteines are believed to be important for the maintenance of the tertiary structure of alpha conotoxins.

**Figure 2:**
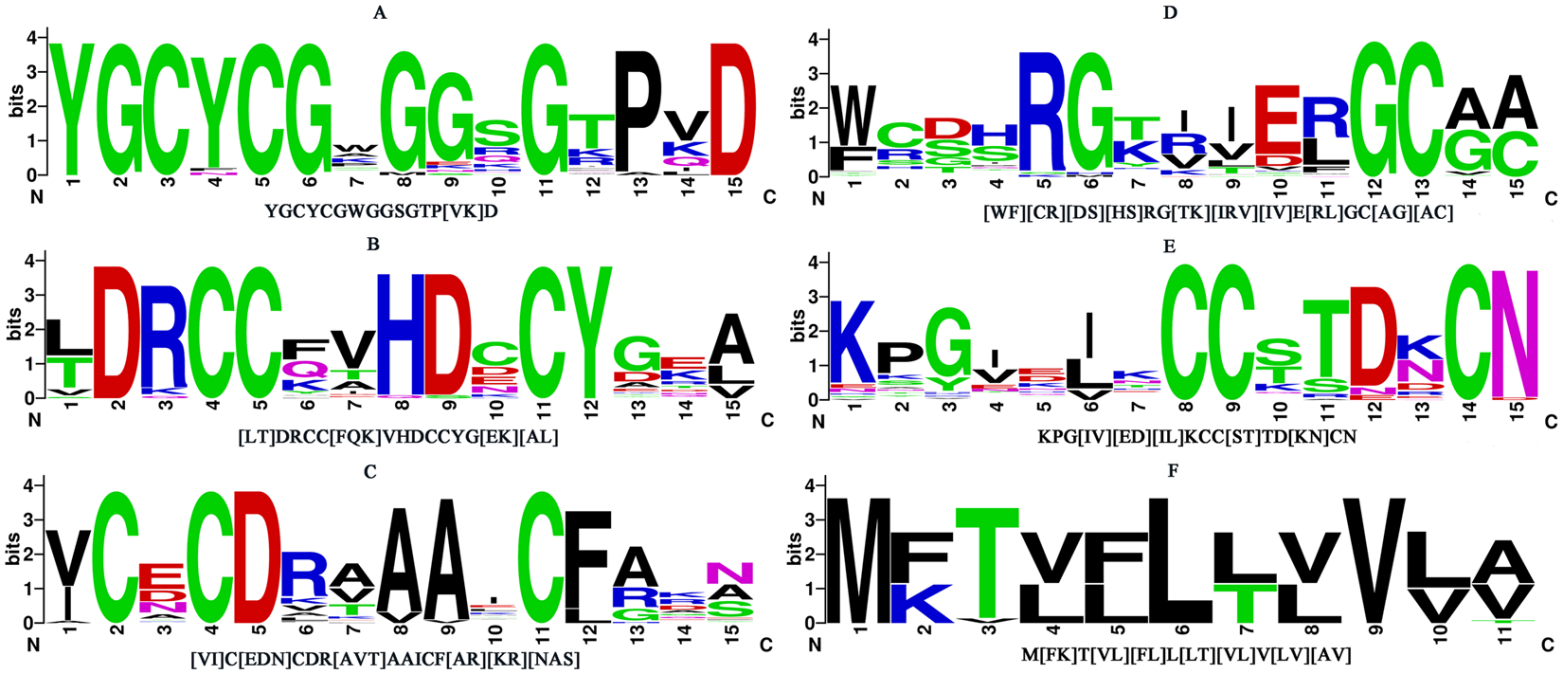
MEME motifs for (**A**) presynaptic neurotoxins motif 1, (**B**) presynaptic neurotoxins motif 2, (**C**) presynaptic neurotoxins motif 3, (**D**) postsynaptic neurotoxins motif 1, (**E**) postsynaptic neurotoxins motif 2, and (**F**) postsynaptic neurotoxins motif 3 in logo format. The regular expression for each MEME motif was shown at the bottom of each figure.

The comparison of MEME motifs (Fig. 2) with Prosite motifs shows that the conserved region from the fourth site to the eleventh site in the presynaptic neurotoxin motif 2 is corresponded to PS000118, this indicate that the presynaptic neurotoxin motif 2 may have the biological function of PS000118; PS000119 is corresponded to the conserved region from the third site to the eleventh site in the presynaptic neurotoxin motif 3; for PS00272, the conserved region from the tenth site to the twenty second site is corresponded to the first site to the twelfth site in the postsynaptic neurotoxin motif 2.

### Prediction of presynaptic and postsynaptic neurotoxins

**Figure 3:**
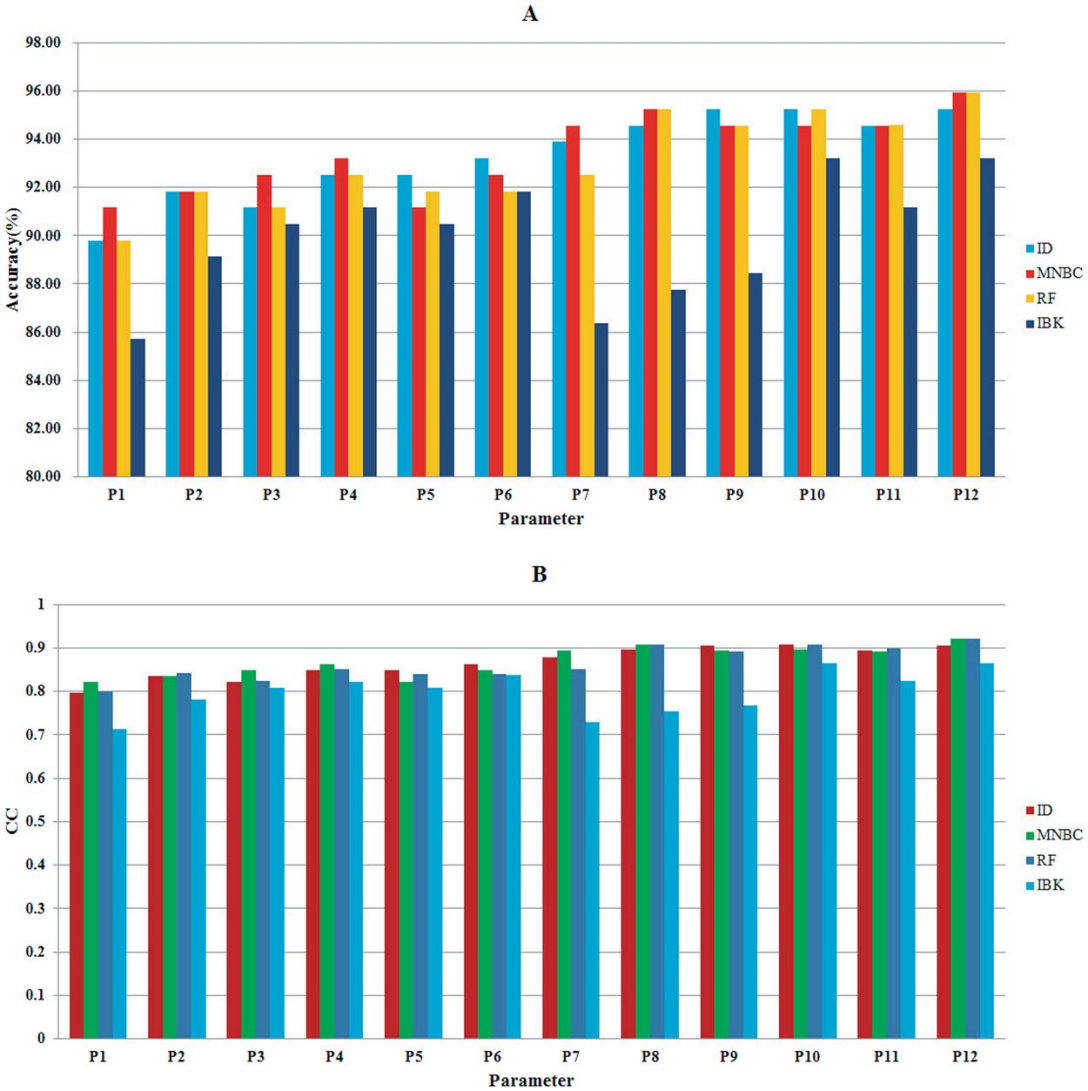
(**A**) Overall predictive accuracies and (**B**) CC values obtained by four different algorithms with 12 parameters.

In order to investigate the influence of different parameters on the prediction quality, 12 different parameters were selected as the input parameters of ID, MNBC, RF, and IBK. The jackknife test results obtained by ID, MNBC, RF, and IBK with 12 different parameters were shown in Tables 1 and 2, Fig. 3A and B.

**Table 1:** Results obtained by ID, MNBC, RF and IBK in identifying presynaptic and postsynaptic neurotoxins with 12 parameters.

	ID				MNBC				RF				IBK			
	Presynaptic		Postsynaptic		Presynaptic		Postsynaptic		Presynaptic		Postsynaptic		Presynaptic		Postsynaptic	
	Sn (%)	Sp (%)	Sn (%)	Sp (%)	Sn (%)	Sp (%)	Sn (%)	Sp (%)	Sn (%)	Sp (%)	Sn (%)	Sp (%)	Sn (%)	Sp (%)	Sn (%)	Sp (%)
P1^a^	88.46	92.00	91.30	87.50	91.03	92.21	91.30	90.00	96.15	82.61	86.21	95.00	88.46	82.61	85.19	86.36
P2	92.31	92.31	91.30	91.30	92.31	92.31	91.30	91.30	98.72	84.06	87.50	98.31	92.31	85.51	87.80	90.77
P3	91.03	92.21	91.30	90.00	93.59	92.41	91.30	92.65	94.87	86.96	89.16	93.75	91.03	89.86	91.03	89.86
P4	93.59	92.41	91.30	92.65	94.87	92.50	91.30	94.03	96.15	88.41	90.36	95.31	93.59	88.41	90.12	92.42
P5	93.59	92.41	91.30	92.65	91.03	92.21	91.30	90.00	97.44	85.51	88.37	96.72	92.31	88.41	90.00	91.04
P6	94.87	92.50	91.30	94.03	93.59	92.41	91.30	92.65	97.44	85.51	88.37	96.72	94.87	88.41	90.24	93.85
P7	97.44	91.57	89.86	96.88	98.72	91.67	89.86	98.41	96.15	88.41	90.36	95.31	84.62	88.41	89.19	83.56
P8	100.0	90.70	88.41	100.0	100.0	91.76	89.86	100.0	100.00	89.86	91.76	100.00	87.18	88.41	89.47	85.92
P9	98.72	92.77	91.30	98.44	98.72	91.67	89.86	98.41	97.44	91.30	92.68	96.92	88.46	88.41	89.61	87.14
P10	100.0	91.76	89.86	100.0	100.0	90.70	88.41	100.0	100.00	89.86	91.76	100.00	92.31	94.20	94.74	91.55
P11	98.72	91.67	89.86	98.41	97.44	92.68	91.30	96.92	97.44	91.43	92.68	96.97	89.74	92.75	93.33	88.89
P12	98.72	92.77	91.30	98.44	100.0	92.86	91.30	100.0	100.00	91.30	92.86	100.00	92.31	94.20	94.74	91.55

^a^Come from^30^ by using Increment of Diversity (ID).

**Table 2:** Overall predictive accuracy and CC values obtained by ID, MNBC, RF and IBK in identifying presynaptic and postsynaptic neurotoxins with 12 parameters.

	ID		MNBC		RF		IBK	
	Presynaptic	Postsynaptic	Presynaptic	Postsynaptic	Presynaptic	Postsynaptic	Presynaptic	Postsynaptic
	Acc (%)	CC	Acc (%)	CC	Acc (%)	CC	Acc (%)	CC
P1^a^	89.80	0.7963	91.16	0.8227	89.80	0.7998	85.71	0.7131
P2	91.84	0.8361	91.84	0.8361	91.84	0.8428	89.12	0.7819
P3	91.16	0.8227	92.52	0.8497	91.16	0.8237	90.48	0.8088
P4	92.52	0.8497	93.20	0.8635	92.52	0.8511	91.16	0.8227
P5	92.52	0.8497	91.16	0.8227	91.84	0.8401	90.48	0.8088
P6	93.20	0.8635	92.52	0.8497	91.84	0.8401	91.84	0.8368
P7	93.88	0.8786	94.56	0.8932	92.52	0.8511	86.39	0.7289
P8	94.56	0.8954	95.24	0.9080	95.24	0.9080	87.76	0.7549
P9	95.24	0.9061	94.56	0.8932	94.56	0.8917	88.44	0.7681
P10	95.24	0.9080	94.56	0.8954	95.24	0.9080	93.20	0.8640
P11	94.56	0.8932	94.56	0.8917	94.59	0.8990	91.16	0.8236
P12	95.24	0.9061	95.92	0.9208	95.92	0.9208	93.20	0.8640

^a^Come from^30^ by using Increment of Diversity (ID).

In this study, when using P12 as the input parameters of ID, MNBC, RF, and IBK for predicting presynaptic and postsynaptic neurotoxins, the overall accuracy of 95.92% and the CC value of 0.9208 were obtained by MNBC and RF, which were the highest overall accuracy and CC value in this study, and were also higher than the predictive results in our previous work^30^. For prediction of presynaptic and postsynaptic neurotoxins, based on the same input parameters, generally speaking, MNBC had the best prediction quality among four algorithms. For example, based on the parameters of P1, P2, P3, P4, P7, P8 and P12, the CC values were 0.8227, 0.8361, 0.8497, 0.8635, 0.8932, 0.9080 and 0.9208 for MNBC, which were 0.0264, 0, 0.0270, 0.0138, 0.0146, 0.0126 and 0.0147 higher than those of ID. The overall accuracies obtained by MNBC were better than or equivalent to those of ID, RF and IBK when using the same parameters. These results clear indicated that MNBC could perform better than three other algorithms for prediction of presynaptic and postsynaptic neurotoxins.

Based on the same algorithm, it was clear that the performances were improved when sequence derived features and motif features were used as input parameters, when compared with other sequence derived features. For ID, when using P2, P3, P4, P5 and P6 as the input parameters, the CC values were 0.8361, 0.8227, 0.8497, 0.8497 and 0.8635, respectively, which were higher than the CC value obtained by P1. Similarly, the higher CC values could also be obtained by MNBC, RF and IBK when using the same parameters. In addition, we found that the predictive results obtained by 19 motifs (13 Prosite motifs and 6 MEME motifs) were better than those obtained by 13 Prosite motifs or 6 MEME motifs in most cases. These results clearly illustrated that the MEME motifs, Prosite motifs and InterPro motifs could significantly improve the predictive power of ID, MNBC, RF and IBK for predicting the presynaptic and postsynaptic neurotoxins.

In this study, the prediction performance was improved by the effective feature selection method when using the same algorithm. Tables 1 and 2 illustrated that the results of the ID, MNBC, RF and IBK with the parameters of P1-P7. Except for the predictive results of IBK, it was clear that higher or equivalent overall accuracy had been obtained by the proposed algorithms with the parameter of P7, when compared with the overall accuracy obtained by the parameters of P1-P6. For example, for the problem of presynaptic and postsynaptic neurotoxins prediction, when P7 was selected as the input parameter, the CC value was 0.8786 for ID, which was 0.0823, 0.0425, 0.0559, 0.0289, 0.0289, and 0.0151 higher than those of P1-P6, respectively. Similarly, except for the predictive results of IBK, the CC value obtained by P7 for MNBC, and RF were also higher than those of P1-P6. These results clearly indicated that MRMR feature selection method was effective and helpful for the prediction of presynaptic and postsynaptic neurotoxins.

For the problem of presynaptic and postsynaptic neurotoxins prediction, as shown in Tables 1 and 2, the sensitivity of presynaptic neurotoxins and the specificity of postsynaptic neurotoxins varied significantly with the parameters, indicating that the prediction results of presynaptic neurotoxins were more correlated with different parameters than the prediction results of postsynaptic neurotoxins. That was because more protein motifs were discovered in the presynaptic neurotoxins than in the postsynaptic neurotoxins. For example, 11 Prosite motifs were discovered by ScanProsite in the presynaptic neurotoxins, however, only 2 Prosite motifs were discovered by ScanProsite in the postsynaptic neurotoxins.

As shown Tables 1 and 2, the best predictive results of ID were obtained by using P10 as the input parameter. In this case, all of the presynaptic neurotoxins were predicted correctly, and 7 postsynaptic neurotoxins were predicted incorrectly. The Animal Toxin database entries numbers of these 7 postsynaptic neurotoxins were AT0001110, AT0000526, AT0002477, AT0000527, AT0000327, AT0002380 and AT0000334, respectively. MEME motifs were not discovered in these postsynaptic neurotoxins, only Prosite motifs and InteroPro motifs were discovered in AT000110 and AT0002380. However, AT000110 and AT0002380 not only belonged to the presynaptic neurotoxins but also belonged to the postsynaptic neurotoxins, and in this case, they were predicted as the presynaptic neurotoxins. Based on these results, we suspected that the motif features may provide an important role in the problem of presynaptic and postsynaptic neurotoxins prediction.

## Discussion

In this paper, in order to predict presynaptic and postsynaptic neurotoxins, 12 different parameters were selected as the input parameters of ID, MNBC, RF, and IBK. The prediction results of the jackknife test were shown in Tables 1 and 2, and Fig. 3. Based on the similar results of different methods presented in Tables 1 and 2, and Fig. 3, we suspected that when using the same parameters, ID, MNBC, RF, and IBK had little impact on prediction results for predicting presynaptic and postsynaptic neurotoxins, and this maybe an intrinsic characteristics of machine learning algorithms which also occurred in the other prediction problems. However, we also found that the input parameters have big impact on prediction results. Taking the ID algorithm as an example, we found that the Acc can increase from 89.80% to 95.24%, and the CC can increase from 0.7963 to 0.9080 for prediction the presynaptic and postsynaptic neurotoxins. Similar improved Acc and CC can also be obtained by other three algorithms. So, the input parameters should have more impact on the prediction results.

In our previous work^30^, for using the same dataset, 78 presynaptic neurotoxins and 69 postsynaptic neurotoxins were predicted by Increment of Diversity (ID), the highest Sn, Sp and CC obtained in our previous work were 88.46%, 92.00% and 0.7963 for presynaptic neurotoxins, and were 91.30%, 87.50% and 0.7963 for postsynaptic neurotoxins, respectively. In this study, we found that, the best Sn, Sp and CC were 100.0%, 92.86% and 0.9208 for presynaptic neurotoxins, and were 91.30%, 100.0%, and 0.9208 for postsynaptic neurotoxins, respectively. Based on these results, we can conclude that the prediction algorithms presented in this study had some advantage over the previous one.

With the increased number of toxins in the public dataset, it is indispensable to develop some reliable methods for classification of presynaptic and postsynaptic neurotoxins. In this study, ID, MNBC, RF, and IBK were applied to classify presynaptic and postsynaptic neurotoxins, a new promising feature representation method was presented by embedding PseAA compositions, MEME motif features, Prosite motif features and InterPro motif features to represent a protein sample. The MRMR feature selection method was also used to select 50 top ranked PseAA compositions to improve the predictive results. In order to obtain the best performance of the proposed algorithms, different kinds of motif features and PseAA compositions were combined and selected as the input parameters of four algorithms. The predictive results presented in this study clearly indicated: (1) MRMR feature selected method, complemented with motif features can significantly improve the prediction quality of neurotoxins; (2) using different parameters would make it possible for algorithms to perform better than the others. The best prediction results were obtained when using 50 PseAA compositions, 46 InterPro motif features and 6 MEME motif features as the input parameters of MNBC and RF. In summary, the above results indicated that ID, MNBC, RF and IBK by using 50 PseAA compositions and biological motif features as the input parameters were reliable for prediction of presynaptic and postsynaptic neurotoxins. We hope that the machine learning algorithms will provide some support for the identification of neurotoxins in the future. The proposed algorithms may become the useful tools in bridging the gap between the huge number of toxins in the public databases and the relatively less number of toxins that have been functionally characterized. As pointed out in Shen and Chou^46^ and demonstrated in a series of recent publications^36, 37, 41, 47–54^, user-friendly and publicly accessible web-servers represent the future direction for developing practically more useful methods that will significantly enhance their impacts^55^, we shall make efforts in our future work to provide a web-server for the analysis method presented in this paper.

## Methods

### Datasets

The dataset generated by Yang and Li was used to estimate the effectiveness of the new prediction methods^30^. The protein sequences in this dataset were downloaded from the Animal Toxin Database (ATDB)^28, 29^. The PISCES^56, 57^ was used to cull the presynaptic and postsynaptic neurotoxin sequences where no two proteins in each dataset had more than 80% sequence identify. In the final dataset, presynaptic neurotoxin dataset consists of 78 protein sequences, and postsynaptic neurotoxin dataset consists of 69 protein sequences.

### Machine learning approaches

In this study, Increment of Diversity (ID)^58^, Multinomial Naive Bayes Classifier (MNBC), Random Forest (RF), and K-nearest Neighbours Classifier (IBK) were used to classify the presynaptic and postsynaptic neurotoxins. The ID algorithm was implemented in the C++ software while the rest of the algorithms were implemented in the Weka package^59^.

### Pseudo amino acid composition

It is very important to select a set of reasonable parameters for protein sequences prediction. As mentioned in previous works, pseudo amino acid composition (PseAAC) is a widely used approach for representation of protein sequences^42, 44, 60–71^, and can be generated by a series powerful webservers developed recently. In this study, according to the concept of the Chou’s PseAA compositions^72–74^, 400 dipeptide compositions were selected as the parameters of our approaches, which were defined in 400-dimension (400-D) space, formulated as:

1 $$Y:\{{y}_{1},{y}_{2},{\rm{\ldots }}{\rm{\ldots }}{y}_{400}\}$$

where *y*_*i*_ (i = 1, 2, 3 …… 400) was the absolute occurrence frequencies of 400 dipeptides.

### Maximum Relevance Minimum Redundancy

In this study, MRMR^34, 35^ was applied on 400 PseAA compositions. After considering both the predictive accuracy and the MRMR score, the top 50 features were selected as the input parameters of the machine learning algorithms, which were defined in a 50-dimension (50-D) space, formulated as:

2 $$Z:\{{z}_{1},{z}_{2},{z}_{3},{\rm{\ldots }}{\rm{\ldots }}{z}_{50}\}$$

### MEME motif features

In this study, the presynaptic and postsynaptic neurotoxin datasets were uploaded to MEME software to conduct motif search^31^. The maximum motif number was set to 3 and the maximum motif length was set to 15. The logo format and the regular expression of these motifs were shown in Fig. 2. Six MEME motifs had been created which were corresponded to the presynaptic neurotoxins and postsynaptic neurotoxins, and the number of motif features was 6. Each element of the vectors represented the presence or absence of a motif in the protein sequences. That was, the corresponded feature value was 1 if a motif was presented; otherwise, it was 0. Consequently, each protein sequence was converted into a 6-dimension (6-D) space, formulated as:

3 $$M:\{{m}_{1},{m}_{2},\cdots \cdots {m}_{6}\}$$

### Prosite motif features

In this study, 11 kinds of Prosite motifs^32^ were found in 78 presynaptic neurotoxin sequences and 2 kinds of Prosite motifs were found in 69 postsynaptic neurotoxin sequences. The total number of motif features was 13. Consequently, each protein sequence was converted into a 13-dimension (13-D) space, formulated as:

4 $$P:\{{p}_{1},{p}_{2},\ldots ,{p}_{13}\}$$

### InterPro motif features

InterPro is an integrated database of protein families, domains and functional sites^33^. In this study, 78 presynaptic neurotoxin sequences and 69 postsynaptic neurotoxin sequences were scanned by InterPro, and 46 functional motifs were found in the neurotoxin datasets. The total number of motif features was 46. Consequently, each protein sequence was converted into a 46-dimension (46-D) space, formulated as:

5 $$N:\{{n}_{1},{n}_{2},\ldots ,{n}_{46}\}$$

### Features for prediction algorithms

In order to improve the prediction accuracy, 400 PseAA compositions, 50 PseAA compositions, 13 kinds of Prosite motifs, 6 kinds of MEME motifs and 46 InterPro motifs were combined. Because the Prosite motifs were contained in the InterPro motifs, so 13 Prosite motifs were not combined with 46 InterPro motifs. P1-P12 indicated 12 kinds of parameters, and these parameters were selected as the input parameters of ID, MNBC, RF, and IBK (Table 3).

**Table 3:** Combination of dipeptide parameters and motif parameters.

Parameters	Number	Description of parameters
P1	400	400 dipeptides
P2	406	400 dipeptides and 6 kinds of MEME motifs
P3	413	400 dipeptides and 13 kinds of Prosite motifs
P4	419	400 dipeptides, 6 kinds of MEME motifs and 13 kinds of Prosite motifs
P5	446	400 dipeptides and 46 kinds of InterPro motifs
P6	452	400 dipeptides, 6 kinds of MEME motifs and 46 kinds of InterPro motifs
P7	50	50 dipeptides selected by MRMR
P8	56	50 dipeptides and 6 kinds of MEME motifs
P9	63	50 dipeptides and 13 kinds of Prosite motifs
P10	69	50 dipeptides, 13 kinds of Prosite motifs and 6 kinds of MEME motifs
P11	96	50 dipeptides and 46 kinds of InterPro motifs
P12	102	50 dipeptides, 46 kinds of InterPro motifs and 6 kinds of MEME motifs

### Evaluation of methods

In this study, in order to roundly estimate the accuracy of our predictor, the sensitivity, specificity, correlation coefficient and overall accuracy were also calculated:

6 $$\{\begin{array}{rcl}Sn & = & \frac{TP}{TP+FN}\\ Sp & = & \frac{TP}{TP+FP}\\ CC & = & \frac{(TP\times TN)-(FP\times FN)}{\sqrt{(TP+FP)\times (TN+FN)\times (TP+FN)\times (TN+FP)}}\\ Acc & = & \sum _{i}\frac{T{P}_{i}}{N}\end{array}$$

where TP denoted the numbers of the correctly recognized positives, FN denoted the number of the positives recognized as negatives, FP denoted the number of the negatives recognized as positives, TN denoted the numbers of correctly recognized negatives, N was the total number of protein sequences.

The set of metrics is valid only for the single-label systems. For the multi-label systems whose existence has become more frequent in system biology^75^ and system medicine^40, 76^, a completely different set of metrics as defined in work of Chou^77^ is needed. In order to take the advantage of using the Chou’s intuitive set of metrics for studying protein signal peptide cleavage site^42, 43, 47–49, 78–82^, the TP, TN, FP, and FN can be represented as follows:

7 $$\{\begin{array}{rcl}TP & = & {N}^{+}-{N}_{-}^{+}\\ TN & = & {N}^{-}-{N}_{+}^{-}\\ FP & = & {N}_{+}^{-}\\ FN & = & {N}_{-}^{+}\end{array}$$

Substituting Eq. (7) into Eq. (6), we can obtain the following metrics:

8 $$\{\begin{array}{rcl}Sn & = & 1-\frac{{N}_{-}^{+}}{{N}^{+}}\\ Sp & = & \frac{{N}^{+}-{N}_{-}^{+}}{{N}^{+}-{N}_{-}^{+}+{N}_{+}^{-}}\\ Acc & = & 1-\frac{{N}_{-}^{+}+{N}_{+}^{-}}{{N}^{+}+{N}^{-}}\\ CC & = & \frac{1-(\frac{{N}_{-}^{+}}{{N}^{+}}+\frac{{N}_{+}^{-}}{{N}^{-}})}{\sqrt{(1+\frac{{N}_{+}^{-}-{N}_{-}^{+}}{{N}^{+}})(1+\frac{{N}_{-}^{+}-{N}_{+}^{-}}{{N}^{-}})}}\end{array}$$

where *N* ^+^ denoted the total numbers of the positives, *N* ^−^ denoted the total numbers of the negatives, ${N}_{+}^{-}$ denoted the number of the negatives incorrectly predicted as positives, and ${N}_{-}^{+}$ denoted the number of the positives incorrectly predicted as negatives. In addition, the jackknife test was also used to validate the prediction power of our algorithms.
